# The Molecular Mechanisms of Imatinib Treatment on Acute Lung Injury in Septic Mice Through Proteomic Technology

**DOI:** 10.1155/jimr/4526375

**Published:** 2025-06-09

**Authors:** Xiao Wang, ZhiQing Zhou, DuanYang Li, BoYang Zhang, XiaoLong Zong, Xue Liang, ZhenYu Li

**Affiliations:** ^1^Department of Emergency Medicine, The Second Hospital of Tianjin Medical University 300211, Tianjin, China; ^2^Department of Clinical Laboratory, The Second Hospital of Tianjin Medical University 300211, Tianjin, China; ^3^Tianjin Key Laboratory of Ionic-Molecular Function of Cardiovascular Disease, Department of Cardiology, Tianjin Institute of Cardiology, The Second Hospital of Tianjin Medical University 300211, Tianjin, China

**Keywords:** acute lung injury, imatinib, proteomics, sepsis

## Abstract

**Background:** Acute lung injury (ALI) is the most common complication of sepsis. Despite considerable progress in the treatment of sepsis, the effective treatment strategies are lacking. A previous study has shown that imatinib reduces the rate of acute pulmonary damage in septic mice; however, the molecular mechanism remains unclear. Therefore, the current study aimed to investigate the potential mechanism by which imatinib alleviates ALI in septic mice.

**Methods:** A septicemia model was established by intraperitoneal injection of lipopolysaccharide (LPS), followed by tail vein injection of imatinib in the treatment group. Enzyme-linked immunosorbent assay (ELISA) was used to detect inflammatory factors, and hematoxylin staining was used to detect pathological injury to the lung tissue. Tandem mass tag (TMT) quantitative labeling technology was used for proteomic sequencing analysis. The main target protein was identified through bioinformatics, and its expression was confirmed using western blotting.

**Results:** We identified 128 differentially expressed proteins were associated with the protective effects of imatinib against septic lung injury. Functional enrichment analysis indicated that these proteins may be related to electron transfer, coagulation, and endothelial cell regulation in the oxidative respiratory chain. Enrichment of the nuclear factor-kappa B (NF-kB) signaling pathway, complement–coagulation cascade, and chemokine signaling pathway was also observed. Additionally, we found that the expression of CCAAT/enhancer-binding protein delta (CEBPD) and pyruvate dehydrogenase kinase 4 (PDK4) increased in the sepsis group but decreased in the imatinib group.

**Conclusion:** Imatinib may reduce ALI in mice with sepsis by participating in oxidative respiratory and inflammatory responses, clotting response-related signaling pathways, and downregulating CEBPD and PDK4 expression.

## 1. Introduction

Sepsis is a life-threatening condition characterized by dysfunction of the host response to infection by bacteria, viruses, and other pathogenic microorganisms, leading to organ dysfunction [1, 2]. Recent studies have shown that the release of a large number of inflammatory factors, production of oxidative stress, and disorders of the coagulation system are the main mechanisms leading to acute lung injury (ALI) [3, 4]. Despite significant progress in the treatment of sepsis, effective and specific treatment strategies for septic lung injury remain lacking [5–7]. Imatinib is the first US Food and Drug Administration (FDA) tyrosine kinase inhibitor and is currently being used to treat chronic lymphocytic tumors and gastrointestinal stromal tumors [8, 9]. Studies have shown that it not only regulates cell proliferation and apoptosis by inhibiting the activity of kinases such as Abl, c-KIT, and PDGFR but also shows significant therapeutic potential in inflammatory diseases and vascular barrier dysfunction [10, 11]. In ALI model, imatinib has been shown to inhibit the expression of proinflammatory cytokines TNF-α, IL-6, and MCP-1 by regulating nuclear factor-kappa B (NF-κB) and MAPK signaling pathways, thereby alleviating lipopolysaccharide (LPS)-induced lung pathological injury and increased vascular permeability. It also maintains the alveolar–capillary barrier function by regulating the structural stability of endothelial cadherin [12–14]. The protective effect of imatinib on the endothelial barrier was further extended to sepsis-related lung injury, especially in the subgroup with significant alveolar epithelial injury combined with systemic inflammation [15, 16]. These studies reveal the multitarget properties of imatinib and its regulatory networks in inflammation, the vascular barrier, and the tumor microenvironment, highlighting its broad applicability as a cross-disease therapeutic strategy. However, the specific molecular mechanism of the therapeutic effect of imatinib has not been fully elucidated, and the therapeutic effect in different pathological backgrounds still needs to be further explored. In recent years, with the development of biomedicine, proteomic research can reveal the essence of life activities and the pathogenesis of diseases, which has been widely used in various fields [17, 18]. Progress has been made in the diagnosis and treatment of sepsis through the integration of online databases. Bian et al. [19] used proteomic techniques to reveal potential mechanisms of hydrogen in the treatment of sepsis, providing a strong basis for its clinical application. Chen et al. [20] used proteomic techniques to elucidate the mechanisms of amino acid metabolism in sepsis. In this study, we used tandem mass tag (TMT) marker-quantified proteomic sequencing to detect relevant differential proteins between sepsis and control groups, sepsis and imatinib groups, and control and imatinib groups. In addition, we combined bioinformatic methods to investigate the functions and signaling pathways of related differentially expressed proteins and selected three differentially expressed proteins for verification by western blotting.

## 2. Methods

### 2.1. Experimentation on Animals

Adult mice, 6–8 weeks old, weighing 18–23 g, were purchased from Spearfish Biotechnology Co. (Beijing, China). The mice were acclimated for 7 days in a ventilated, quiet controlled environment (temperature 20–23°C), ensuring adequate food and water. A total of 36 mice were used in the experiment and randomly divided into three groups (*n* = 12): control (C), LPS (L), and LPS + imatinib (M) groups. All the experiments were approved by the Animal Conservation and Use Committee of the Second Hospital of Tianjin Medical University (TMUaMEC2023002). An ALI model of endotoxin-poisoned mice was established by intraperitoneal injection of 15 mg/kg LPS (*Escherichia coli* 0111: B4, Sigma-Aldrich, USA). The treatment group received an injection 60 mg/kg imatinib into the tail vein 0.5 h before modeling. After 24 h of modeling, mice from each group were sacrificed, and blood was collected using the eye blood sampling method. The supernatant was then extracted for enzyme-linked immunosorbent assay (ELISA). Three mice from each group were randomly selected for pathological analysis. The remaining tissues were used for proteomic analysis. Animals that died during modeling were excluded from the analysis, and experiments were performed as permitted by the project.

### 2.2. ELISA-Based Analysis of Mice Serum

Serum levels of inflammatory factors were analyzed using the LEGENDplex Mouse Inflammation Panel (13-plex) (740446; BioLegend, USA). All protocols were performed according to the manufacturer's instructions.

### 2.3. Hematoxylin and Eosin (H&E) Staining

The upper lung tissue was fixed with 4% paraformaldehyde for 48 h, paraffin-embedded sections were obtained, routine H&E staining was performed, and pathological changes were observed under a light microscope.

### 2.4. Immunofluorescence Staining

Lung tissues were paraffin-fixed and incubated overnight at 37°C. Then, these lung sections were deparaffinized and hatched with 3% hydrogen peroxide for 15 min. The slices were heated at microwave treatment and then naturally cooled for 40 min. After performing antigen retrieval, the samples were blocked with 1% BSA for 0.5 h and add monoclonal antibody at 4°C overnight. Lastly, homologous fluorescent or biotin-labeled secondary antibodies were incubated with the lung tissues for 2 h at 37°C. The images were captured using a microscope.

### 2.5. Protein Immunoblotting

Expression levels of CCAAT/enhancer-binding protein delta (CEBPD), pyruvate dehydrogenase kinase 4 (PDK4), and UCP2 (uncoupling protein 2) were detected by western blotting. Lung tissue proteins were extracted, and their concentrations were determined using the bicinchoninic acid assay (BCA) (23225, Thermo Scientific, USA) method. Protein samples were separated by sodium dodecyl sulfate–polyacrylamide gel electrophoresis (SDS-PAGE) and electrodeposited onto polyvinylidene fluoride (PVDF) membranes (IPVH00010, Sigma-Aldrich, USA). The membranes were blocked with a tris-buffered saline containing 0.1% Tween-20 (TBST, B548117-0500, Sango Biotech, USA) and 5% skim milk (D8340, Solarbio, CH) at room temperature for 1 h. After washing, anti-CEBPD, anti-UCP2, and anti-PDK4 antibodies were added, and the membranes were incubated at 4°C overnight. The membranes were washed three times with TBST, incubated with the secondary antibody labeled with catalase for 1 h, and washed again three times with TBST. Finally, substrate chemiluminescence was performed, and the expression level of target protein was determined by calculating the ratio of target protein band's grayscale intensity to that of β-actin. Primary and secondary antibodies used are listed in Table 1.

### 2.6. Tandem Mass Spectrometric Labeling

Fifty micrograms protein was extracted from each sample, and samples from different groups were diluted to the same concentration and volume using a pyrolysis solution.

The samples were then mixed with 5 mM dithiothreitol (DTT, 1064272, Adamas-beta, USA) and incubated for 30 min at 55°C. The mixture was then cooled on ice until reaching room temperature. The corresponding volume of iodoacetamide was added to the samples at a concentration of 10 mM, mixed at room temperature, and kept in the dark for 15 min. A sixfold volume of acetone precipitated protein was added to the solution and then stored at −20°C for more than 4 h or overnight. The precipitated mixtures were collected at 4°C and centrifuged at 8000× *g* for 10 min, and acetone was evaporated for 2–3 min. One hundred microliter tetraethylammonium bromide (TEAB) (T7408-500 ML, Sigma–Aldrich, USA) (200 mM) precipitated solution and 1 mg/mL tosyl phenylalanyl chloromethyl ketone (TPCK)–treated trypsin (20230, Thermo Scientific, USA) of 1/50 sample mass were added and digested overnight at 37°C. The lyophilized samples were stored at −80°C after freeze-drying. A 100 mM TEAB was added to the freeze-dried samples, shaken, and mixed evenly in a 1.5 mL Eppendorf (Ep) tube. TMT reagent was removed from the refrigerator to balance it to room temperature. Anhydrous acetonitrile was then added to re-dissolve it, and the mixture was centrifuged. TMT reagent was added to the samples and mixed thoroughly. The mixture was then left at room temperature for 1 h, and 5% hydroxylamine was added to stop the reaction. The mixture was then stored at −80°C after freeze-drying.

### 2.7. Separation by Reversed-Phase Chromatography

Liquid chromatography: Agilent 1100 high-performance liquid chromatography (HPLC). Chromatographic column: Agilent Zorbax Extend – C18 narrow diameter column, 2.1 × 150 mm, 5 μm. Detection wavelength: UV 210 nm. Mobile phase A: ACN-H2O (2:98, v/v), pH was adjusted to 10 with ammonia. Mobile phase B: ACN-H2O (90:10, v/v), pH was adjusted to 10 with ammonia. Velocity: 300 μ L/min. Gradient elution conditions: 0–8 min, 98% A; 8–8.01 min, 98%–95% A; 8.01–30 min, 95%–80% A; 30–43 min, 80%–65% A; 43–53 min, 65%–55% A; 53–53.01 min, 55%–10% A; 53.01–63 min, 10% A; 63–63.01 min, 10%–98% A; and 63.01–68 min, 98% A. Samples were collected for 8–54 min, with the eluant collected into centrifuge tube numbered 1–15 every other minute. Subsequently, the samples were collected repeatedly for tubes numbered 1–15. The collected samples were dried and frozen for MS.

### 2.8. Liquid Chromatography With Tandem Mass Spectrometry (LC-MS/MS)

Chromatographic conditions: Samples were separated using a flow rate of 300 nL/min on an Acclaim PepMap RSLC column, 75 μm × 50 cm (RP-C18, Thermo Fisher, USA). Mobile phase A: ACN-H2O-FA (99.9 : 0.1, v/v). Mobile phase B: ACN-H2O-FA (80:19.9:0.1, v/v/v). Gradient elution conditions: 0–50 min, 2%–28% B; 50–60 min, 28%–42% B; 60–65 min, 42%–90% B; and 65–75 min, 90% B. Conditions for MS: mass resolution of Grade I MS was set to 60,000, automatic gain control value was set to 3e6, and maximum injection time was set to 50 ms. MS scan was set to full scanning charge to mass ratio m/z range of 350–1500, and MS/MS scan was performed for 20 of the highest peaks. All MS/MS spectrum collection and use of data dependent on positive ion mode was completed. Collision energy was set to 32, resolution of MS/MS to 45000, automatic gain control to 2e5, maximum injection time of ions to 80 ms, and dynamic exclusion time to 30 s.

### 2.9. Database Retrieval

The data were analyzed using Proteome Discoverer 2.4.1.15, and the search library sequence file was Uniprot-*Mus musculus*-10090-2023. fast2.1.a.

### 2.10. Bioinformatics Analysis

After retrieving the original data from the database, Score Sequest HT > 0, unique peptide ≥1 was screened to ensure protein expression without null values. Trusted proteins were obtained after median standardization and log2 logarithmic conversion of the screened protein. The differences between the two standards were calculated based on trusted proteins. Fold change (mean value of the experimental group: mean value of the control group) was used to assess the multiple changes in expression levels among samples, and the *p*-value calculated by the *t*-test showed the significance of differences among samples. Differential screening conditions were as follows: fold change ≥1.5 or fold change ≤1/1.5 and *p* < 0.05. Differential protein genes were annotated using the gene ontology (GO) database to enrich the top 10, and the top 50 were enriched by the Kyoto Encyclopedia of Genes and Genomes (KEGG) database. The differential proteins that increased in the sepsis group but decreased in the imatinib group were screened for protein interaction network analysis.

### 2.11. Statistic Analysis

SPSS 25.0 software was used for analysis in this study. One-way analysis of variance (ANOVA) was used for comparison between groups, and Tukey multiple comparison test was used to analyze the comparison between the groups. Statistical significance was set at *p* < 0.05.

## 3. Results

### 3.1. Model Validation

We observed that the mice in group C (control group) were active, with shiny fur, normal appetite, and agile movements. Compared to group C, the mice in group L (LPS group) exhibited signs of depression, cuddled and curled up posture, reduced activity, poor appetite, increased urination and defecation, ocular discharge, and sluggish movements. Mice in group M (LPS + IMA group) also showed huddling, fear of cold, and decreased appetite, but it was significantly reduced than that in group L.

**Figure 1:**
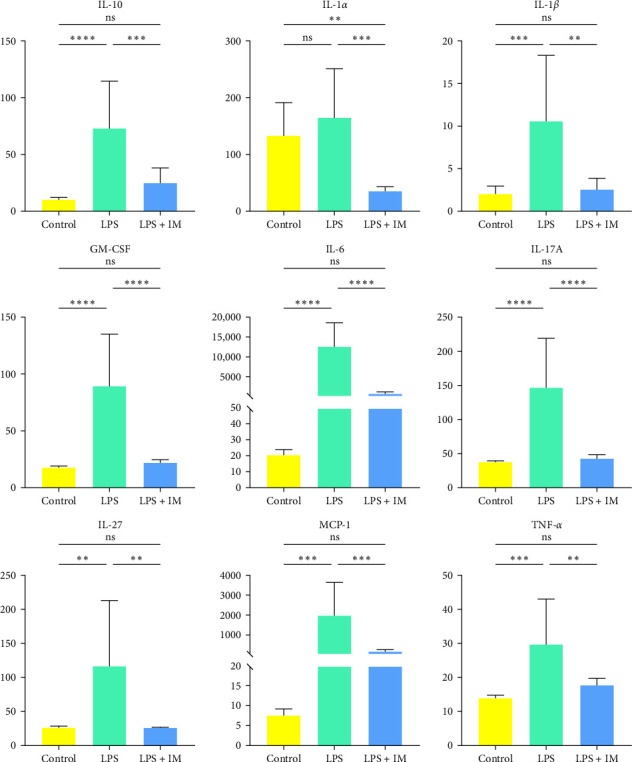
The expression of various inflammatory factors in each group. *⁣*^*∗∗*^*p* < 0.01, *⁣*^*∗∗∗*^*p* < 0.001, and *⁣*^*∗∗∗∗*^*p* < 0.0001.

A total of 11 inflammation-related factors were identified, and the results showed significant changes in the following inflammatory markers: (GM-CFS/IL-1α/IL-1β/IL-6/IL-10/MCP-1/TNF-α/IL-17A/IL-27). These inflammatory markers increased significantly and were statistically significant in the sepsis group (LPS) compared with the control group (C) and decreased in the treatment group (IMA) compared with the sepsis group (LPS) (Figure 1).

**Figure 2:**
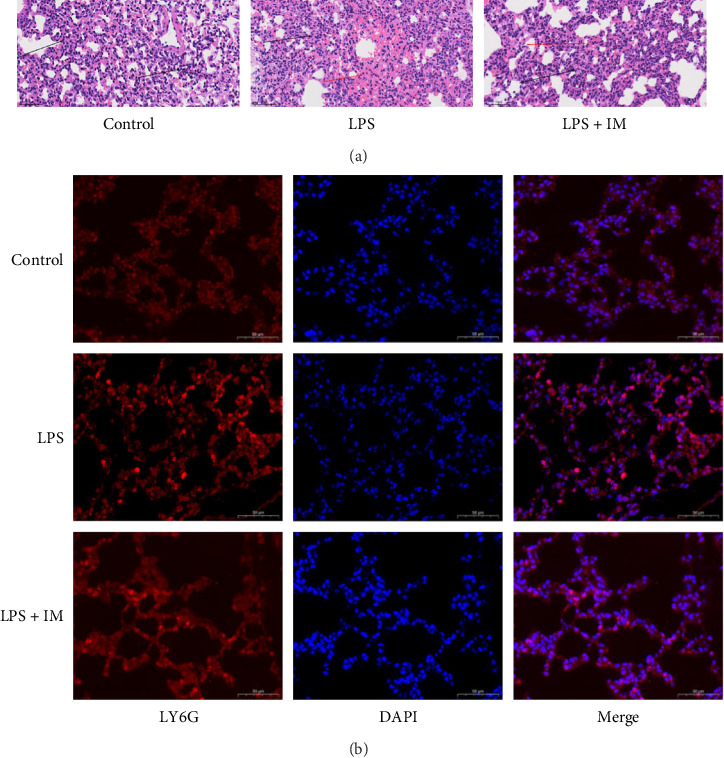
Pathological changes of lung tissue in the three groups and pathological test results of each group (A). Representative images of Ly6g immunofluorescence staining of lung sections of the three groups of mice: red, Ly-6 G; blue, DAPI; scale bar, 50 μm (B).

Pathological examination revealed that, in comparison to the control group, pulmonary alveolar size of the mice in the sepsis group (LPS) was different, the focal alveolar interval was widened, blood vessels were dilated and congested, and inflammatory cell proliferation was obvious. Mice in the imatinib intervention group (IMA) showed varying alveolar sizes, widening of the focal alveolar interval (mild to moderate), vasodilation, and moderate congestion, which were less pronounced than those observed in the L group (LPS) (Figure 2A).

The results of immunofluorescence staining showed, compared with the control group, LY6G colocalization was significantly enhanced in LPS group, indicating significant pulmonary neutrophil infiltration in LPS group. After imatinib treatment, LY6G colocalization was relatively reduced, indicating that the degree of neutrophil infiltration was lower than that in LPS group (Figure 2B).

These results suggest that the mouse ALI model has been successfully established.

### 3.2. General Characteristics of Proteins

**Figure 3:**
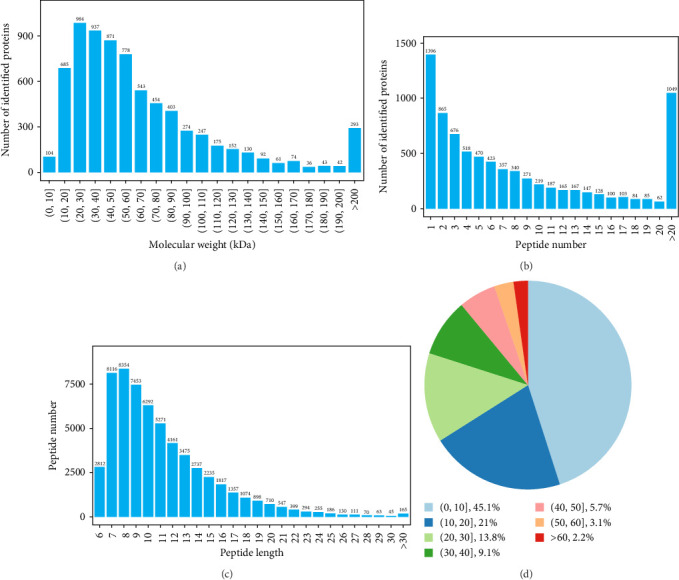
Protein counts of different molecules (A). Qualitative distribution of peptides corresponding to each protein (B). The length of the peptide corresponding to each protein (C). The peptide coverage (%) (D).

Protein counts for every molecule are illustrated in Figure 3A. The sequence of peptides for each of the qualitative proteins is represented by a list of proteins, with the majority of them corresponding to at least two peptides, as illustrated in Figure 3B; Figure 3C illustrates the distribution of the various peptides associated with each qualitative protein, with the majority ranging between 7 and 20 amino acids, and a peptide was found, which covered the majority of the proteins at a rate of 20% (Figure 3D).

### 3.3. Screening for Differential Proteins

**Figure 4:**
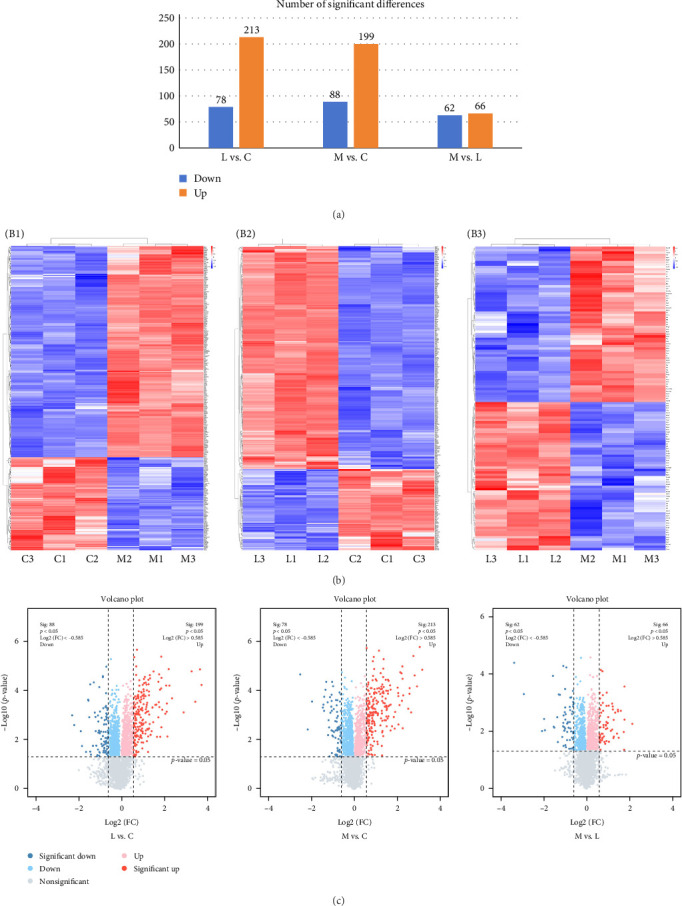
The abscissa represents the comparative group, and the ordinate represents the quantity of the various proteins. If there are three or more ANOVA groups in an entry, one column indicates how many proteins are present in the group, and the color is irrelevant (A). Thermal maps for the various proteins, protein expression is grouped by the quantity of the protein expressed. Red indicates high-expressed protein, blue indicates low-expressed protein, and every row indicates the level of expression of various proteins (B (B1–B3)). The horizontal coordinate of the volcanic chart is log2 (FC). The vertical axis is −log10 (*p*), with a larger deviation as the vertical is from 0. In the diagram, the blue and red spots denote the upregulation and downregulation of the proteins, respectively, with a deeper color denoting a more pronounced difference and a gray spot representing a protein having a *p*-value less than 0.05 (C). C, control group; L, LPS group; M, imatinib treatment group.

The results revealed 7378 proteins in these three groups. Using this screening method, 706 proteins were identified, including 128 proteins associated with sepsis and imatinib, 291 proteins associated with sepsis, and 287 proteins associated with imatinib (Figure 4A). The thermal maps for the various proteins in each group are illustrated in Figure 4B (B1–B3), while the corresponding volcano maps for the various proteins are illustrated in Figure 4C.

### 3.4. Functional Enrichment Analysis of Differential Proteins

**Figure 5:**
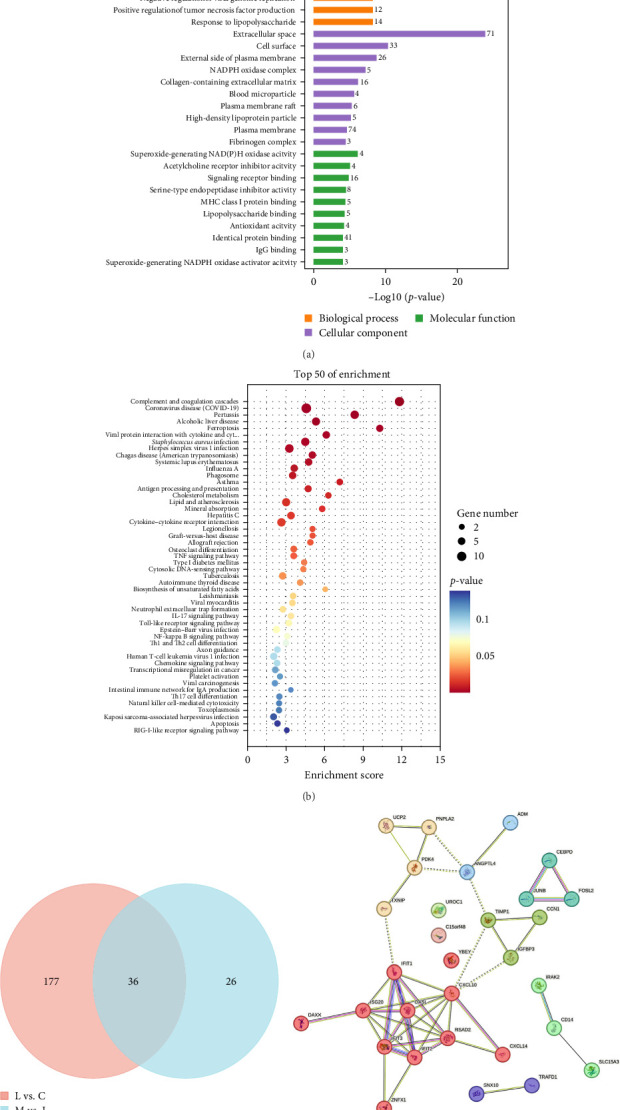
The horizontal coordinate represents the −log10 *p*-value, the vertical coordinate represents the GO entry name, and the numbers on the bars indicate the number of differential proteins annotated to each entry (A). The score in the horizontal coordinates is the enrichment score, and the pathway information on the vertical coordinates indicate the top 20 enriched results. Objects with bigger bubbles have a greater number of different proteins, and their color ranges from blue to red. The lower the concentration *p*-value, the more significant the result (B). Various colors indicate a variety of groups. The figures in this diagram show how many proteins are superimposed on one another and how many are specific for each group (C). Interconnected protein networks composed of differential proteins (D).

Outcomes of the GO analysis indicated the top 20 enriched results at a molecular level as small molecule binding, pheromone incorporation, growth factor, T-cell receptor incorporation, serine endopeptidase inhibitor activity, hematoxylin incorporation, transforming growth factor receptor, and MHC class I proteins; at a cellular level as extracellular interstitial collagen-containing fine extracellular matrix, outer side of plasma membrane pieces, blood particles, complement component C1q complex, fibrinogen complex, platelet granules, extracellular region, cell surface, and component C1 assemblies; and at a biological level as acute temporal response, bacterial response, response to styrenes, blood coagulation, fibrin thrombosis, positive regulation of allogeneic cells, cellular response to sodium ions, defensive response to viruses, fibrinolysis, negative regulatory role of endothelial cell apoptosis, and innate immune response (Figure 5A). KEGG database was used to enrich the differentially expressed proteins associated with the pathway. The first 50 results are shown in Figure 5B. We found that these proteins were involved in complement and coagulation cascade, TNF signaling pathway, extracellular trap of neutrophil, IL-17 signaling pathway, NF-κb signaling pathway, toll receptor signaling pathway, chemokine signaling pathway, platelet activation, differentiation of TH1 and TH2 cells, RIG-I receptor signaling pathway, apoptosis, and cytochrome receptor interaction signaling pathway.

Simultaneously, we screened 36 related differential proteins whose expression increased in the sepsis and control groups but decreased in the imatinib and sepsis groups (Figure 5C). We also performed functional interactive network analysis based on the STRING database (Figure 5D).

### 3.5. Verification of Differential Protein

**Figure 6:**
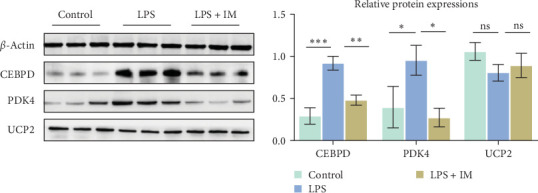
Western blotting detected the expression levels of CEBPD, PDK4, and UCP2. *⁣*^*∗*^*p* < 0.05, *⁣*^*∗∗*^*p* < 0.01, *⁣*^*∗∗∗*^*p* < 0.001.

The quantitative proteomic findings were validated through western blotting; CEBPD, PDK4, and UCP2 were screened and confirmed by western blot analysis. CEBPD and PDK4 were significantly upregulated in the sepsis group (L group), but downregulated in the imatinib group (M group), consistent with our sequencing results. However, the expression of UCP2 was not significantly altered across the three groups of mice, which contradicted our sequencing results (Figure 6).

## 4. Discussion

Sepsis is one of the most fatal diseases globally and is usually associated with multiorgan dysfunction. ALI is a common sepsis complication. Currently, ALI treatment primarily focuses on symptomatic management, yet the mortality rate among patients with sepsis remains high. In this study, we found that treatment with imatinib could decrease pulmonary pathological damage and related inflammatory factors in mice. Using the TMT quantitation assay, we identified 128 differentially expressed proteins among patients with sepsis and those treated with imatinib. GO labeling and enrichment, KEGG pathway analysis, and STRING Protein Interactive Net were used to analyze the differences.

GO analysis showed that imatinib-associated differentially expressed proteins were mainly involved in the coagulation reaction, delivery of the oxidative respiratory chain, and regulation of the cellular endothelium. When sepsis occurs, the invasion of various bacteria and pathogenic microorganisms leads to the expression of endothelial cell tissue factors, which initiate the coagulation system, leading to dysregulation of the coagulation and fibrinolytic systems, resulting in the deposition of fibrin and the occurrence of ALI [21]. Severe sepsis causes a large number of inflammatory factors to form an inflammatory cascade amplification effect, releases reactive oxygen species (ROS), and impairs mitochondrial function. These effects lead to the destruction of the electron transport chain of oxidative respiration and affect cellular oxygen transport [22]. Endothelial cell damage is a key factor in the development of septic lung injury [23], and Aman et al. [24] experimentally found that imatinib attenuated the disruption of the endothelial barrier of the human umbilical vein by thrombin. Therefore, imatinib may protect against ALI in septic mice by attenuating damage to endothelial cells, reducing impairment of mitochondrial function, improving oxygen transport, and reversing imbalances in the coagulation and fibrinolytic systems.

In the pathway analysis, we found that relevant differential proteins mediate in the NF-κB signaling pathway, complement and coagulation cascade response, chemokine signaling pathway, cytochrome–cytochrome receptor interactions, and RIG-I signaling pathway. Previous studies have also demonstrated that imatinib attenuates ALI in septic mice by modulating the NF-κB signaling pathway [25]. A study by François found that imatinib inhibited the activation of thrombin to attenuate lung injury. This finding suggests that imatinib may attenuate the damage effects of disturbances in the coagulation system on lung tissue by inhibiting the activation of thrombi [26], which is consistent with our analysis of the sequencing results. Additionally, as mentioned earlier, mitochondrial damage and disruption of the oxidative respiratory chain are also key changes in the development of sepsis. Further, cytochrome–cytochrome receptor is a key factor in the oxidative respiratory chain; therefore, we consider that imatinib attenuates the ALI in septic mice by participating in the electron transfer process in the oxidative respiratory chain, which then improves the oxygen supply and other related responses. Moreover, RIG-I activates interferon and inflammatory cytokine production [27], suggesting that imatinib may reduce the release of inflammatory factors by mediating the involvement of upstream RIG-I receptors in signaling pathways such as NF-κB.

Significant enrichment of the three clusters identified in the protein interaction network analysis suggested that imatinib was primarily involved in the regulation of inflammation and clotting-related responses.

Finally, we screened three related differentially expressed proteins using comparative analysis and verified that the results at the protein level were consistent with the sequenced protein level.

CEBPD belongs to the C/EBP group, which is also called the C/EBPδ pathway in cellular differentiation, inflammation, and metabolism. In hypoxia, HIF1α and HIF2α can stimulate CEBPD expression, which is activated by activation of PIK3/AKT pathway and IL-6Rα, which then triggers down STAT3 [28, 29]. The activation of PIK3/AKT and STAT3 has been shown to amplify inflammation in a cascade and induce apoptosis. Our sequencing analysis indicated a significant increase in CEBPD in patients with sepsis and a decline in therapy, suggesting that imatinib can suppress inflammation and apoptosis through direct downregulation of CEBPD.

PDK4 belongs to PDK, which is expressed in the mitochondria; PDC is a pyruvate dehydrogenase complex, which plays an important role in oxytricarboxylic acid cycle. PDK4 inhibits PDC activity, thereby preventing pyruvate from entering the tricarboxylic acid cycle [30]. Sepsis causes an imbalance in oxygen supply/demand, impairs oxygen use, and consequently elevates lactate levels, which are important factors in the poor prognosis of patients with sepsis [31]. Our histology indicated that PDK4 was elevated in patients with sepsis but was reduced when imatinib was administered. This result suggests that imatinib might reduce the effect on PDC by downregulating PDK4 expression, thereby attenuating the inhibitory effect on PDC and oxidative response and improving oxygen supply in sepsis, consequently attenuating ALI in sepsis.

UCP2 is a member of the mitochondrial disjunction and is involved in uncoupling in the mitochondrial proton delivery chain. LPS-induced generation of excess ROS leading to impaired mitochondrial function can lead to UCP2 activation. UCP2 activation can inhibit the generation of ROS through negative feedback, thus protecting mitochondrial function, and may act through the UCP2/AMPK axis [32, 33]. Our protein analysis indicated that UCP2 was elevated in patients with severe sepsis and imatinib-treated patients. However, our western blot results showed no statistically significant differences among the three groups. On the one hand, since the mice were sacrificed 24 h after modeling, the expression of UCP2 may not have or just started to increase at this time. On the one hand, since the mice were killed 24 h after modeling, UCP2 expression may not have increased or was just beginning to increase at this time. On the other hand, we consider proteomic sequencing as an auxiliary tool, whose coincidence rate cannot reach 100%, and there may be false positive results. Therefore, the effect of UCP2 on lung injury in sepsis requires further studies.

The intervention effect of imatinib may be due to its synergistic regulation of three protein molecules: blocking the TLR4/CEBPD signal axis by inhibiting c-Abl kinase activity and downregulating PDK4 expression to restore mitochondrial energy metabolism. In the future, it is necessary to further reveal the functional coupling characteristics of key proteins in the pathogenesis of ALI, so as to provide theoretical basis for precise treatment strategies targeting multitarget interactive networks.

## 5. Conclusion

In summary, we have identified 128 differentiated proteins related to the protection of imatinib from septic pulmonary damage by TMT quantitation assay. Through functional enrichment and analysis, we obtained that imatinib may reduce sepsis lung injury by ameliorating sepsis-induced impairment of mitochondrial function, modulating the electron transfer process in the oxidative respiratory chain, and improving oxygen supply. In addition, imatinib downregulated CEBPS, PDK4, and UCP2 by expression to alleviate ALI in septic mice. Our findings improve more potential understanding of the pathogenesis of sepsis lung injury and provide new insights into the potential molecular mechanisms by which imatinib attenuates sepsis lung injury. What we have discovered is that we may be able to better understand the underlying mechanism of septic pulmonary damage and to shed light on the possible molecular mechanisms through which imatinib can attenuate the pulmonary damage in sepsis.

## Figures and Tables

**Table 1:** Primary and secondary antibodies used in western blot.

Antibodies	Dilution	Solvent
Primary antibodies		
Rabbit anti-CEBPD (ab245214)	1:1000	TBST
Rabbit anti-PDK4 (ab97931)	1:1000	TBST
Rabbit anti-UCP2 (ab214938)	1:1000	TBST
Mouse anti-β-actin (ab8226)	1:1000	TBST
Secondary antibodies		
Goat anti-rabbit IgG-HRP (ab7090)	1:5000	TBST
Goat anti-mouse IgG-HRP (ab7040)	1:5000	TBST

## Data Availability

The authors confirm they have included a data availability statement in their manuscript (BioProject: PXD050204).

## Nomenclature

LPS: Lipopolysaccharide
NF-κB: Nuclear factor-kappa B
CEBPD: CCAAT/enhancer-binding protein delta
PDK4: Pyruvate dehydrogenase kinase 4
FDA: Food and Drug Administration
ELISA: Enzyme-linked immunosorbent assay
H&E: Hematoxylin and eosin
BCA: Bicinchoninic acid assay
PVDF: Polyvinylidene fluoride
DTT: Dithiothreitol
TPCK: Tosyl phenylalanyl chloromethyl ketone
TMT: Tandem mass tag
HPLC: High-performance liquid chromatography
LC: Liquid chromatography
MS: Mass spectrometry
GO: Gene ontology
KEGG: Kyoto Encyclopedia of Genes and Genomes
ANOVA: Analysis of variance
ROS: Reactive oxygen species.

## References

[1] Bellani G., Laffey J. G., Pham T. (2016). Epidemiology, Patterns of Care, and Mortality for Patients With Acute Respiratory Distress Syndrome in Intensive Care Units in 50 Countries. *JAMA*.

[2] Blay J.-Y., Kang Y.-K., Nishida T., von Mehren M. (2021). Gastrointestinal Stromal Tumours. *Nature Reviews Disease Primers*.

[3] Cohen P. (2002). Protein Kinases—The Major Drug Targets of the Twenty-First Century?. *Nature Reviews Drug Discovery*.

[4] Fan E. K. Y., Fan J. (2018). Regulation of Alveolar Macrophage Death in Acute Lung Inflammation. *Respiratory Research*.

[5] Fein A. M., Calalang-Colucci M. G. (2000). Acute Lung Injury and Acute Respiratory Distress Syndrome in Sepsis and Septic Shock. *Critical Care Clinics*.

[6] Ferguson N. D., Kacmarek R. M., Chiche J. D. (2004). Screening of ARDS Patients Using Standardized Ventilator Settings: Influence on Enrollment in a Clinical Trial. *Intensive Care Medicine*.

[7] Huang M., Cai S., Su J. (2019). The Pathogenesis of Sepsis and Potential Therapeutic Targets. *International Journal of Molecular Sciences*.

[8] Singer M., Deutschman C. S., Seymour C. W. (2016). The Third International Consensus Definitions for Sepsis and Septic Shock (Sepsis-3). *JAMA*.

[9] Stapleton R. D., Wang B. M., Hudson L. D., Rubenfeld G. D., Caldwell E. S., Steinberg K. P. (2005). Causes and Timing of Death in Patients With ARDS. *Chest*.

[10] Akgun-Cagliyan G., Cort-Donmez A., Kilic-Toprak E., Altintas F. (2022). Verbascoside Potentiates the Effect of Tyrosine Kinase Inhibitors on the Induction of Apoptosis and Oxidative Stress via the Abl-Mediated MAPK Signalling Pathway in Chronic Myeloid Leukaemia. *Experimental and Therapeutic Medicine*.

[11] Kim J. L., Lee D. H., Jeong S. (2019). Imatinib-Induced Apoptosis of Gastric Cancer Cells Is Mediated by Endoplasmic Reticulum Stress. *Oncology Reports*.

[12] Liu Y., Li D., Zhang T. (2025). Effect of Imatinib on Lipopolysaccharide-Induced Acute Lung Injury and Endothelial Dysfunction Through the P38 MAPK and NF-*κ*B Signaling Pathways In Vivo and In Vitro. *Respiratory Physiology & Neurobiology*.

[13] Rizzo A. N., Sammani S., Esquinca A. E. (2015). Imatinib Attenuates Inflammation and Vascular Leak in a Clinically Relevant Two-Hit Model of Acute Lung Injury. *American Journal of Physiology-Lung Cellular and Molecular Physiology*.

[14] Stephens R. S., Johnston L., Servinsky L., Kim B. S., Damarla M. (2015). The Tyrosine Kinase Inhibitor Imatinib Prevents Lung Injury and Death After Intravenous LPS in Mice. *Physiological Reports*.

[15] de Brabander J., Duijvelaar E., Schippers J. R. (2022). Immunomodulation and Endothelial Barrier Protection Mediate the Association Between Oral Imatinib and Mortality in Hospitalised COVID-19 Patients. *European Respiratory Journal*.

[16] Rizzo A. N., Belvitch P., Demeritte R., Garcia J. G. N., Letsiou E., Dudek S. M. (2020). Arg Mediates LPS-Induced Disruption of the Pulmonary Endothelial Barrier. *Vascular Pharmacology*.

[17] Rozanova S., Barkovits K., Nikolov M., Schmidt C., Urlaub H., Marcus K. (2021). Quantitative Mass Spectrometry-Based Proteomics: An Overview. *Methods in Molecular Biology*.

[18] Pimienta G., Heithoff D. M., Rosa-Campos A. (2019). Plasma Proteome Signature of Sepsis: A Functionally Connected Protein Network. *Proteomics*.

[19] Bian Y., Qin C., Xin Y. (2018). Itraq-Based Quantitative Proteomic Analysis of Lungs in Murine Polymicrobial Sepsis With Hydrogen Gas Treatment. *Shock*.

[20] Chen Q., Liang X., Wu T. (2022). Integrative Analysis of Metabolomics and Proteomics Reveals Amino Acid Metabolism Disorder in Sepsis. *Journal of Translational Medicine*.

[21] Huang L., Zhang L., Ju H. (2015). Stanniocalcin-1 Inhibits Thrombin-Induced Signaling and Protects From Bleomycin-Induced Lung Injury. *Scientific Reports*.

[22] Joffre J., Hellman J. (2021). Oxidative Stress and Endothelial Dysfunction in Sepsis and Acute Inflammation. *Antioxidants & Redox Signaling*.

[23] Joffre J., Hellman J., Ince C., Ait-Oufella H. (2020). Endothelial Responses in Sepsis. *American Journal of Respiratory and Critical Care Medicine*.

[24] Aman J., van Bezu J., Damanafshan A. (2012). Effective Treatment of Edema and Endothelial Barrier Dysfunction With Imatinib. *Circulation*.

[25] Mokhtari D., Li T., Lu T., Welsh N., Ryffel B. (2011). Effects of Imatinib Mesylate (Gleevec) on Human Islet NF-KappaB Activation and Chemokine Production In Vitro. *PLoS ONE*.

[26] François D., Arocas V., Venisse L. (2018). Hematopoietic Protease Nexin-1 Protects Against Lung Injury by Preventing Thrombin Signaling in Mice. *Blood Advances*.

[27] Onomoto K., Onoguchi K., Yoneyama M. (2021). Regulation of RIG-I-Like Receptor-Mediated Signaling: Interaction Between Host and Viral Factors. *Cellular & Molecular Immunology*.

[28] Mao X. G., Xue X. Y., Lv R. (2023). CEBPD Is a Master Transcriptional Factor for Hypoxia Regulated Proteins in Glioblastoma and Augments Hypoxia Induced Invasion Through Extracellular Matrix-Integrin Mediated EGFR/PI3K Pathway. *Cell Death & Disease*.

[29] Chen J. C., Alvarez M. J., Talos F. (2014). Identification of Causal Genetic Drivers of Human Disease Through Systems-Level Analysis of Regulatory Networks. *Cell*.

[30] Agil A., El-Hammadi M., Jiménez-Aranda A. (2015). Melatonin Reduces Hepatic Mitochondrial Dysfunction in Diabetic Obese Rats. *Journal of Pineal Research*.

[31] Sterling S. A., Puskarich M. A., Shapiro N. I. (2013). Characteristics and Outcomes of Patients With Vasoplegic Versus Tissue Dysoxic Septic Shock. *Shock*.

[32] Zheng G., Lyu J., Liu S. (2015). Silencing of Uncoupling Protein 2 by Small Interfering RNA Aggravates Mitochondrial Dysfunction in Cardiomyocytes Under Septic Conditions. *International Journal of Molecular Medicine*.

[33] Mao J.-Y., Su L.-X., Li D.-K., Zhang H.-M., Wang X.-T., Liu D.-W. (2021). The Effects of UCP2 on Autophagy Through the AMPK Signaling Pathway in Septic Cardiomyopathy and the Underlying Mechanism. *Annals of Translational Medicine*.

[34] Wang X. W., Zhou Z.-Q. Z., Li D.-Y. (2024). The Molecular Mechanisms of Imatinib Treatment on Acute Lung Injury in Septic Mice Through Proteomic Technology.

